# Supplementary material for: Back to Nature: Medicinal Plants as Promising Sources for Antibacterial Drugs in the Post-Antibiotic Era
Source: Plants (Basel). 2023 Aug 28;12(17):3077. doi: 10.3390/plants12173077 (PMC10490416; doi:10.3390/plants12173077)
Supplement: Supplementary file 1 [file plants-12-03077-s001.zip › plants-2421877-supplementary.pdf]

Supplementary materials

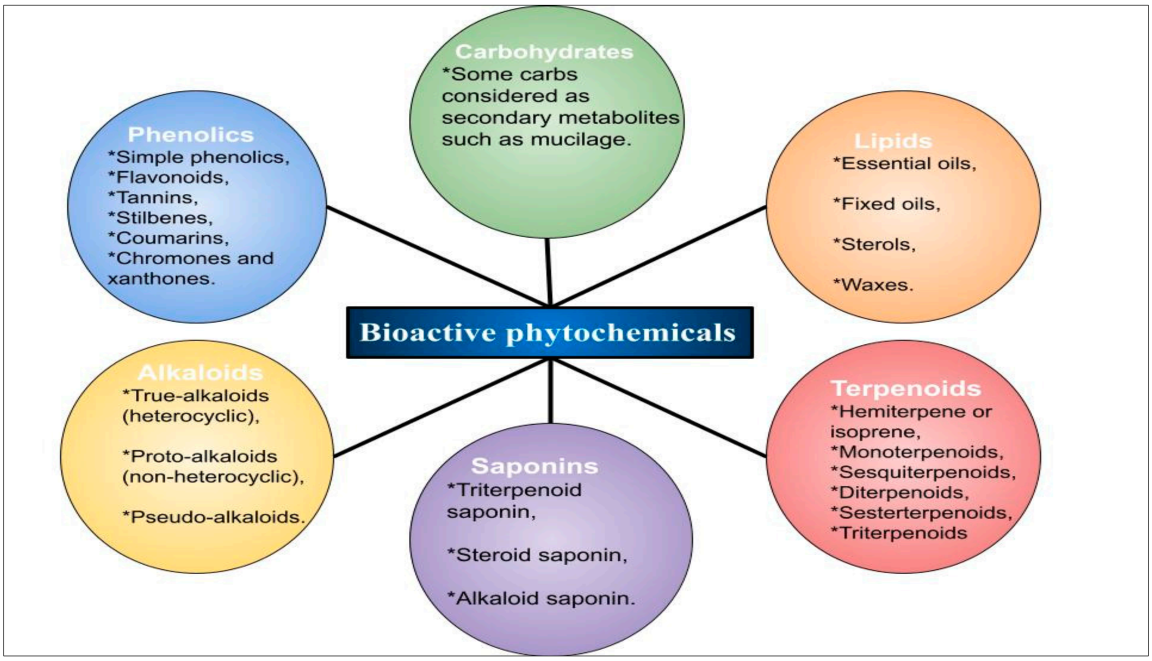

Figure S1. The main classes of bioactive phytochemicals that may include antibacterial agents

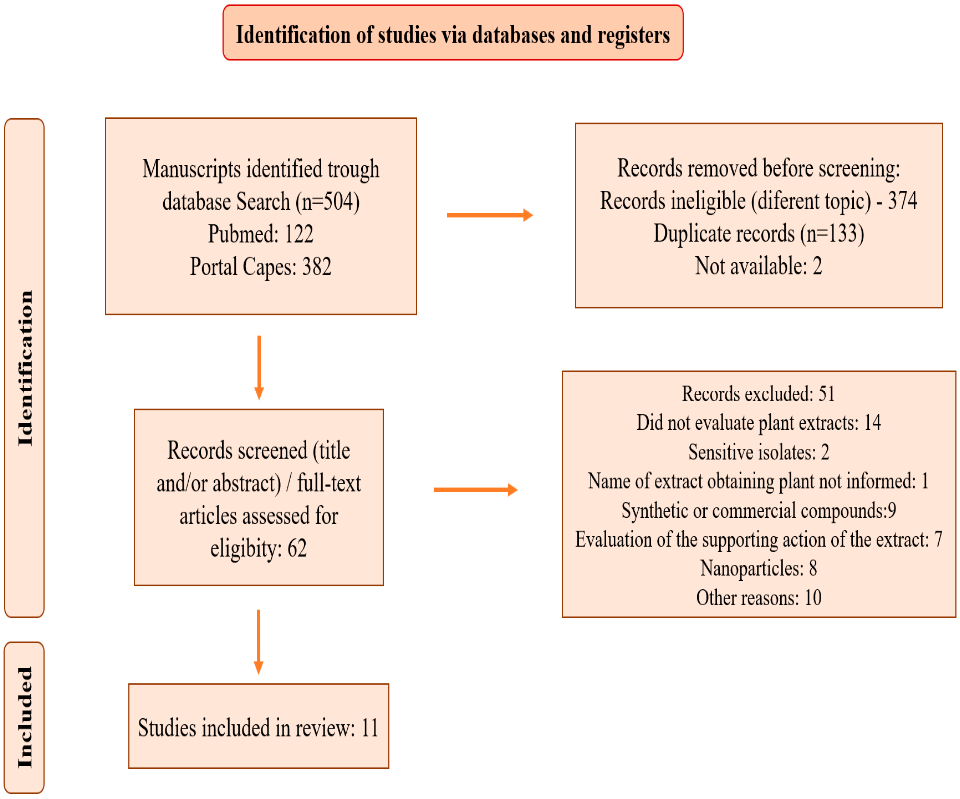

Figure S2: Flowchart of the selection of articles to be evaluated in the current review
